# Electronic Structure and *d*-Band Center Control Engineering over Ni-Doped CoP_3_ Nanowall Arrays for Boosting Hydrogen Production

**DOI:** 10.3390/nano11061595

**Published:** 2021-06-17

**Authors:** Jing Qi, Tianli Wu, Mengyao Xu, Dan Zhou, Zhubing Xiao

**Affiliations:** Henan Key Laboratory of Photovoltaic Materials, Henan University, Kaifeng 475004, China; jingqi_henu@163.com (J.Q.); mengyaoxu_henu@163.com (M.X.); hxzhoud@163.com (D.Z.)

**Keywords:** transition-metal phosphide, electrocatalyst, hydrogen-evolution reaction, density-functional theory

## Abstract

To address the challenge of highly efficient water splitting into H_2_, successful fabrication of novel porous three-dimensional Ni-doped CoP_3_ nanowall arrays on carbon cloth was realized, resulting in an effective self-supported electrode for the electrocatalytic hydrogen-evolution reaction. The synthesized samples exhibit rough, curly, and porous structures, which are beneficial for gaseous transfer and diffusion during the electrocatalytic process. As expected, the obtained Ni-doped CoP_3_ nanowall arrays with a doping concentration of 7% exhibit the promoted electrocatalytic activity. The achieved overpotentials of 176 mV for the hydrogen-evolution reaction afford a current density of 100 mA cm^−2^, which indicates that electrocatalytic performance can be dramatically enhanced via Ni doping. The Ni-doped CoP_3_ electrocatalysts with increasing catalytic activity should have significant potential in the field of water splitting into H_2_. This study also opens an avenue for further enhancement of electrocatalytic performance through tuning of electronic structure and *d*-band center by doping.

## 1. Introduction

Electrocatalytic splitting of water into hydrogen is one of the most simple, eco-friendly, and promising approaches to solving gradual environmental pollution, the energy crisis, and issues related to global warming [1,2,3,4,5]. The key to practical application is to develop highly efficient and inexpensive non-noble-metal electrocatalysts as substitutes for noble-metal electrocatalysts, which are expensive and scarce [6,7,8,9,10]. Transition-metal phosphides (TMPs) have been reported recently as promising hydrogen-evolution-reaction (HER) electrocatalysts because they have a structure similar to hydrogenase [11,12,13]. However, since they suffer from sluggish kinetics, the electrocatalytic activity of TMPs is far less than that of noble-metal electrocatalysts with close to zero overpotential. Therefore, increasingly greater attention has been paid to the improvement of TMPs catalytic efficiency via reduction of the overpotential and enhancement of the electrocatalytic HER durability.

Among such TMPs, Co phosphides (Co_2_P, CoP, CoP_2_, and CoP_3_) have attracted much research interest owing to their fine electrocatalytic performance and stability [14,15,16,17]. Moreover, heteroatom doping can tune the electronic structures and energy-level modulation so as to tune the HER electrocatalytic capability and to reduce the hydrogen adsorption free energy in catalysts [18,19,20]. Thus far, Fe-, Mn-, Cu-, and N-doped Co_2_P and CoP have been studied extensively [21,22,23,24], with the results indicating that the doped CoP and Co_2_P exhibit enhanced electrochemical performance compared with undoped electrocatalysts. In our recent work, CoP_3_ was shown to exhibit outstanding electrochemical activity due to having more active sites (P*) compared with Co_2_P and CoP [25,26]. However, to meet the requirement for industrial applications, several issues must still be addressed, including the following: (i) improvement of the electrocatalytic activity in CoP-based electrocatalysts, and (ii) elucidation of the real active species for HER activity, which has not yet been realized [27,28,29]. Therefore, modifying the electronic structures and energy levels and developing the morphologies of the TMP catalysts are crucial factors for improving their catalytic activity, which can be an achievable yet challenging task.

Motivated by the above analysis, an electronic band structure and *d*-band central control scheme of CoP_3_ nanowall arrays (NWAs) is proposed to accelerate the HER process in acidic and alkaline media, simply via vacuum-phosphorizing precursors in a sealed quartz tube. In virtue of the rapid gaseous transfer and diffusion during the electrocatalytic procedure endowed by the 3D interconnected porous structure, the NWAs exhibit highly efficient catalytic HER activity, exceeding the efficiency of both dopant-free catalysts and the majority of the previously reported non-noble-metal electrocatalysts. Moreover, with combined spectroscopic studies and theoretical calculations, it is confirmed that the enhanced electrocatalytic activity originates from the change of both the electronic band structure and *d*-band center following Ni doping, whereby the downward movement of the *d*-band center from the Fermi level weakens the H binding strength. This study opens an avenue for further enhancing the electrocatalytic activity by tuning the electronic structures and *d*-band center via doping.

## 2. Materials and Methods

### 2.1. Materials and Chemicals

Co(NO_3_)_3_·6H_2_O, NaH_2_PO_4_, Na_2_HPO_4,_ H_2_SO_4_, KOH, urea, and NH_4_F were purchased from Aladdin Ltd. (Shanghai, China). Nafion ethanol solution (5 wt.%) and commercial Pt/C (20 wt.%) electrocatalysts were purchased from Adamas. All other chemical reagents used were of analytical grade sans additional purification. CC was provided by Shanghai Hesen Corp.

### 2.2. Synthesis of Ni-Doped CoP_3_ NWAs

**Figure 1:**
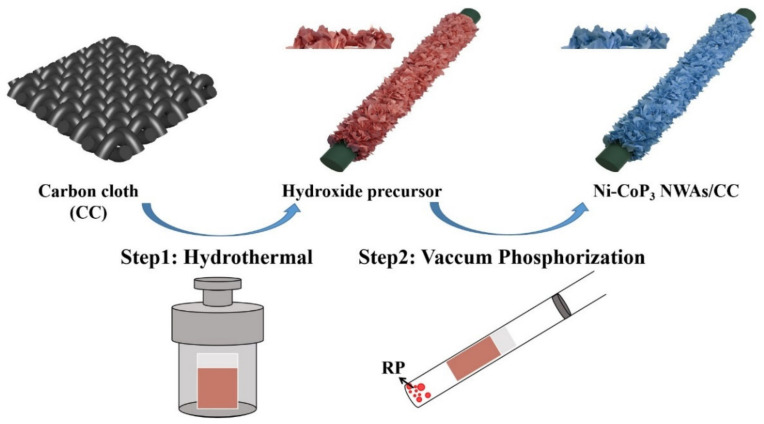
Schematic of synthesis procedure of Ni-doped CoP_3_ nanowall arrays.

The Ni-doped CoP_3_ NWAs on CC were prepared using the procedure illustrated in Figure 1. The hydroxide precursor on the CC was prepared via the hydrothermal method, the precursor and P were then sealed in a quartz tube by vacuum, and finally annealed at 750 °C to yield porous Ni-doped CoP_3_ NWAs/CC. These processes are schematically elucidated in Figure 1. In a typical synthesis procedure, to prepare the hydroxide precursor on CC, Co(NO_3_)_2_·6H_2_O (1.1640 g), Ni(NO_3_)_2_·6H_2_O (0.116 g), NH_4_F (0.3720 g), and urea (1.200 g), dissolved in 80 mL of deionized water (DI), were energetically stirred and transferred into a Teflon™-lined stainless-steel autoclave (50 mL) after 40 min. Meanwhile, a slice of CC (3 cm × 2 cm) was cleaned sequentially with acetone, DI, and ethanol solution. The autoclave with the mixture solution, in which the treated CC was immersed, was sealed and heated at 120 °C for 6 h in an electric oven. The CC with hydroxide precursor was washed thoroughly with DI water when the electric oven cooled down to room temperature. Then, to prepare Ni-doped CoP_3_ NWAs on CC, a piece of CC (0.6 cm × 1.5 cm) with hydroxide precursor and 30 mg of red phosphorus were vacuum-sealed (10^−4^ Pa) into a quartz tube, which was then heat-treated at 750 °C for 5 h in a furnace.

### 2.3. Materials Characterization

X-ray-diffraction (XRD) analysis with Cu *K*α radiation at 40 kV and 50 mA was carried out using a diffractometer (PANalytical X’pert, PANalytical B.V., Almelo, Holland). The samples’ morphology, size, composition, and structure were characterized using field-emission scanning electron microscopy (FE-SEM, TESCAN MARI3, Tescan Ltd., Brno, Czech) and transmission electron microscopy (TEM, FEI TECNAI G2 F20, FEI Co., Hillsboro, OR, USA), combined with energy-dispersive X-ray (EDX) spectroscopy. The samples were measured by XPS using an Al *K*α (1486.6 eV) X-ray source on a spectrometer (ESCALAB 250XI, Thermo Fisher Scientific Co., Waltham, MA, USA). The C 1*s* peak at 284.6 eV was selected for energy calibration to eliminate sample charging during analysis. The adsorption–desorption isotherms of nitrogen were acquired on a surface-area analyzer (JW-BK200A, Beijing JWGB Sci. & Tech. Co., Ltd., Beijing, China) in which all samples were deaerated at 100 °C prior to measurement. Hydrogen adsorption experiment was characterized by adding 30 mg of active materials into 50 mL of 1 M hydrochloric acid solution. After the solution was stirred for 2 h, the centrifugation was carried out and the pH change of the supernatant was measured to determine the amount of hydrochloric acid adsorbed by the active materials.

### 2.4. Electrochemical Measurements

Electrochemical workstations (CHI 660E, Chenhua Co., Shanghai, China) were used to analyze the HER polarization curves in 0.5 M H_2_SO_4_ at room temperature. A saturated calomel electrode (SCE) and graphite rod were used as the reference electrode and counter electrode, respectively. The SCE electrode was calibrated relative to the reversible hydrogen electrode (RHE). Linear-sweep-voltammetry (LSV) data were collected at a scanning rate of 2 mV s^−1^. The time dependence of the catalytic current during the electrolysis of the catalyst in 0.5 M H_2_SO_4_ was measured at potentiostatic voltage. Electrochemical impedance spectroscopy (EIS) were made in the 0.01–10^5^ Hz frequency range.

### 2.5. Theoretical Calculation

The detailed calculations were based on published literature [30]. In general, first-principles calculation was performed using the Vienna ab initio simulation package (VASP) based on the density-functional theory (DFT) and the electron–ion interactions were conducted by generalized gradient approximation Perdew–Burke–Eznerhof (GGA-PBE) method. All the models were forced to a self-consistent accuracy of 10^−5^ eV. The kinetic energy cut-off of 500 eV for the plane-wave basis restriction and K-points were taken under Monkhorst-Pack for Brillouin-zone integration. The periodic images of the slab were separated by 15 Å of vacuum. To calculate the electronic properties and hydrogen evolution activity, a 5 × 5 × 1 supercell was used. The HER characteristics were evaluated by ΔGH* defined as ΔG_H*_ = ΔE_H_ + ΔE_ZPE_ − TΔS_H_, where ΔE_H_, ΔE_ZPE_, and ΔS_H_ are the differential hydrogen adsorption energy, change in the zero point energy, and entropy between adsorbed hydrogen and molecular hydrogen in the gas phase, respectively, and T is the temperature.

## 3. Results and Discussion

**Figure 2:**
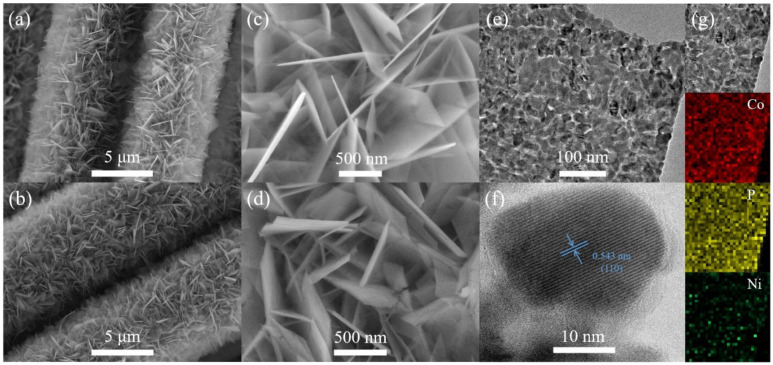
(**a**,**c**) SEM images of Ni-doped cobalt precursor NWAs/CC. (**b**,**d**) SEM images of Ni-CoP_3_-7 NWAs/CC. (**e**) TEM and (**f**) HRTEM images of Ni-CoP_3_-7 NWAs/CC. (**g**) TEM and corresponding elemental mapping images of Ni, Co, and P for Ni-CoP_3_-7 NWAs/CC.

The Ni-doped CoP_3_ NWAs/CC were fabricated using the procedure illustrated in Figure 1. Briefly, the hydroxide precursor on CC was first prepared via a hydrothermal method, the subsequent annealing under high vacuum at 750 °C gave rise to the porous Ni-doped CoP_3_ NWAs/CC. SEM and TEM were employed to observe the morphologies of the Co-based precursor NWAs/CC and CoP_3_ NWAs/CC. The SEM images (Figure 2a,b) show that the Ni-doped Co-based precursor has an assembly walls-like morphology characterized by a smooth surface approximately 20–30 nm in thickness that evenly covers the CC surfaces. At a heating rate of 2 °C min^−1^ for topotactic phosphorization, the Ni-doped CoP_3_ NWAs maintain the three-dimensional (3D) NWA structures and original morphology of Ni-doped precursor (Figure 2c). The fact that all surfaces of the Ni-doped CoP_3_ NWAs are rough is confirmed both by HRSEM images (Figure 2d) and TEM images (Figure 2e), demonstrating that the as-synthesized CoP_3_ NWAs/CC have high porosity and a large surface area, which is verified by subsequent specific surface-area measurements. Such a rough and porous surface can significantly increase the number of active sites, thereby increasing the electrochemical surface area. The active sites of the electrocatalyst are enhanced, and the specific surface area increased, by the porous structure. The HRTEM image shown in Figure 2f reveals a clear crystal-lattice fringe with an interplanar spacing of 0.543 nm that corresponds to the (110) plane of Ni-CoP_3_, indicating that the prepared Ni-CoP_3_ nanowalls are highly crystalline. In addition, elemental mapping shows that Co, P, and Ni are uniformly distributed in the nanowall of Ni-CoP_3_ after doping (Figure 2g) at an atom ratio of Ni:Co:P = 0.063:1:2.94 (Figure S1).

**Figure 3:**
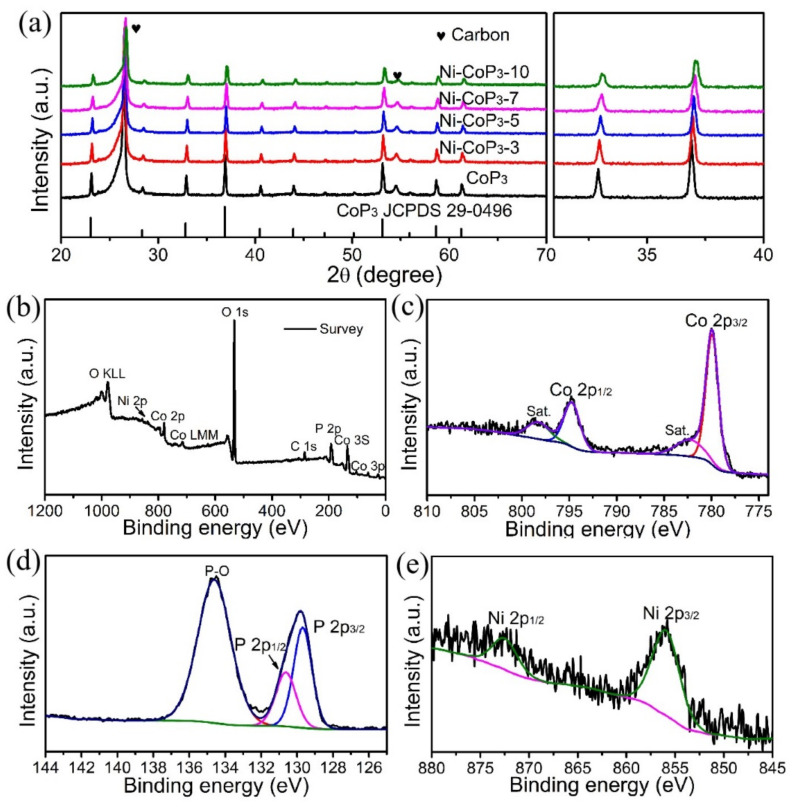
(**a**) XRD patterns and XPS (**b**) survey, (**c**) Co 2*p*, (**d**) P 2*p*, and (**e**) Ni 2*p* spectra of Ni-CoP_3_-7 NWAs/CC sample.

The XRD pattern (Figure 3a) for the as-phosphorized products shows that diffraction peaks are indexed to the planes of CoP_3_, which agreed well with the CoP_3_ standard pattern (JCPDS Card No. 29-0496). In addition, no peaks related to any impurities are present, except two peaks from the CC (JCPDS Card No. 26-1080), which demonstrate the high phase purity of the products. The diffraction peaks of Ni-CoP_3_ NWAs/CC shift to a large angle by degrees with increment of Ni concentration (Figure 3a), which reflect that the replacement Co (atomic radius 152 pm) by Ni (149 pm) in the CoP_3_ cause the deflation of the CoP_3_ lattice. However, because of their similar atomic sizes, this atom replacement do not change the crystal structure of CoP_3_. After XRD refinement, the lattice parameter of Ni-CoP_3_-7 is calculated to be a = b = c = 7.703 Å, which is slightly lower than that of CoP_3_ (a = b = c = 7.711 Å), meaning that the lattice parameter decreases with the increment of Ni substitution degree. In short, the morphology and composition of Ni-doped CoP_3_ NWAs were controlled by high vacuum and a slow ramping rate during the phosphorization process.

The N_2_ adsorption/desorption isotherm of Ni-CoP_3_-7 NWAs exhibits a large specific surface area of ca. 73 m^2^ g^−1^, which is slightly smaller than that of CoP_3._ This may be due to the slight decrease in the crystallization quality derived from Ni doping (see Figure S2 in the Supplementary Material). The large specific surface area confers more catalytic active sites, thus promoting transfer of electrons to the catalytic active sites and a reaction with the osmotic electrolyte to produce hydrogen. The pore size distribution curve of the Ni-CoP_3_-7 NWAs shows a broad peak ranging from 2 to 25 nm, with a dominant peak at ca. 3 nm and a relatively high pore volume as compared to that of the CoP_3_ NWAs. Additionally, the H^+^ adsorption amount (based on HCl) is 7.13 g g^−1^ following stable adsorption in the H^+^ adsorption experiments (Figure S3), proving that large amounts of protons can accumulate on the surface of the Ni-CoP_3_-7 NWAs/CC, which may be conducive to the excellent HER activity of the Ni-CoP_3_-7 NWAs/CC.

The composition and elemental valence states of as-prepared samples were measured by XPS. In Figure 3b, the XPS survey spectrum of Ni-CoP_3_-7 NWAs/CC indicates the coexistence of Ni, Co, and P elements in the sample. The Co 2*p* spectrum shows the characteristic peaks for Co 2*p*_3/2_ (Figure 3c), the binding energy (BE) of which is at 780.0 eV, certifying the existence of Co^3^^+^ [31,32]. In addition, the Co 2*p*_1/2_ peak is located at 794.7 eV, accompanied by a satellite peak at 798.3 and 782.3 eV [21,22]. The characteristic peaks at 129.7 and 130.5 eV are assigned to P 2*p*_1/2_ and P 2*p*_3/2_ (Figure 3d), respectively [33]. The peak of oxidized P species at 134.6 eV was also observed due to the exposure of the samples to air [34]. It is worth mentioning that the XPS spectrum of Co 2*p* in Ni-CoP_3_-7 are mostly identical to that of pure CoP_3_ [14]. However, the P 2*p* peak in the Ni-CoP_3_-7 NWs/CC shifted to low energy, compared with that of pure CoP_3_ [14]. A greater negative shift of BEs signified that the sites of P can easily gather the electrons to join the catalytic reaction, and the electrocatalytic performance can correspondingly be enhanced [35]. In Figure 3e, the weak peaks at 852.9 (Ni 2*p*_3/2_) and 869.7 eV (Ni 2*p*_1/2_) belong to Ni-P bonds, indicating the successful doping of Ni atoms. In short, XRD and XPS analysis results confirm the successful synthesis of Ni-doped CoP_3_ NWAs/CC.

**Figure 4:**
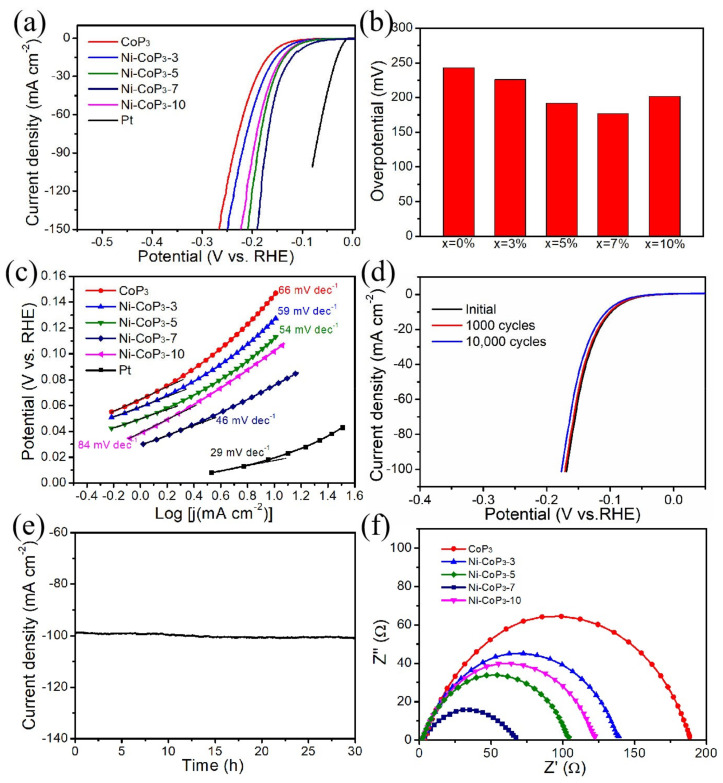
(**a**) HER performance of as-prepared Ni-CoP_3_ NWAs/CC with different Ni concentrations and commercial Pt/C catalyst in 0.5 M H_2_SO_4_. (**b**) HER overpotentials for delivering current densities of 100 mA cm^−2^ and (**c**) corresponding Tafel slopes for Ni-CoP_3_ NWAs/CC with different Ni concentrations. (**d**) LSV curves of Ni-CoP_3_-7 before and after 10,000 cycles of cyclic-voltammetry scans. (**e**) Time dependence of current density for Ni-CoP_3_-7 at static overpotential of 172 mV for 30 h. (**f**) EIS for Ni-CoP_3_ NWAs/CC with different Ni concentrations.

To study the HER catalytic activity and stability of the Ni-doped CoP_3_ NWAs/CC, electrochemical evaluations were carried out in a three-electrode system with iR-compensation under identical conditions. To activate the catalysts before recording data, all test electrodes were cycled 50 times between −0.6 and 0 V versus RHE at 10 mV s^−1^. Figure 4a plots the LSV curves at a scan rate of 2 mV s^−1^ in 0.5 M H_2_SO_4_ electrolyte. The bare CC shows negligible HER activity (Figure S4). The commercial Pt/C electrode exhibits highly efficient catalytic activity with an onset overpotential close to zero for the HER, while the Ni-CoP_3_-7 NWs/CC exhibits the best catalytic performance toward the HER, only acquiring overpotentials of −95 and −177 mV at 10 and 100 mA cm^−2^, respectively (Figure 4a,b). The electrocatalytic performance of Ni-CoP_3_-7 toward HER at high current density is indeed better than the previously reported HER electrocatalysts as shown in Table S1. The Tafel slope (Figure 4c) values are 66, 59, 54, 46, and 84 mV dec^−1^ for pure CoP_3_, Ni-CoP_3_-3, Ni-CoP_3_-5, Ni-CoP_3_-7, and Ni-CoP_3_-10, respectively, further revealing the super-catalytic behavior of Ni-CoP_3_-7 NWAs/CC. The exchange current density of the Ni-CoP_3_-7 is calculated to be −0.217 mA cm^−^^2^, which is larger than most of the non-noble-metal HER electrocatalysts (Table S1). Another important contributor to the electrocatalytic activity is the electrochemically active surface area, which is estimated by the specific double-layer capacitance (C_dl_) at the solid–electrolyte interface. As shown in Figure S5, the Ni-CoP_3_-7 NWAs show a C_dl_ of 108 mF cm^−^^2^, which is larger than that of CoP_3_, indicating that Ni-CoP_3_-7 NWAs have higher active surface area than that of CoP_3_ NWAs. The HER performance of Ni-CoP_3_ NWAs was further evaluated based on turnover frequency (TOF). Figure S6 exhibits the polarization curves of various electrocatalysts normalized by the active sites. Both the TOF and Tafel slope indicate that the intrinsic performance of CoP_3_ can be enhanced by doping. Moreover, Figure 4d clearly depicts that the final polarization curve (after 1000 and 10,000 cycles) is slightly different from the original. In addition, the I-t curve also shows little loss for the HER after 30 h (Figure 4e), confirming the superior electrocatalytic stability of Ni-CoP_3_-7 toward the HER. To better understand the enhanced HER performance, impedance measurements were made for all Ni-doped CoP_3_ NWAs/CC. Figure 4f shows the corresponding Nyquist plots: the lower resistance of Ni-CoP_3_-7 NWAs/CC suggests that doping changes the electronic structure of the materials, resulting in a decrease in its impedance. In this case, the tuning of electronic structures can influence the surface-adsorption strength of intermediate species and the activation energy for the catalytic step in electrocatalysis, in agreement with the LSV curves and Tafel slopes. In addition, this conclusion is also confirmed by calculation of electron band structure and Gibbs free energy below. The excellent HER activity of the Ni-CoP_3_-7 NWAs/CC make it a promising catalyst for water-splitting.

**Figure 5:**
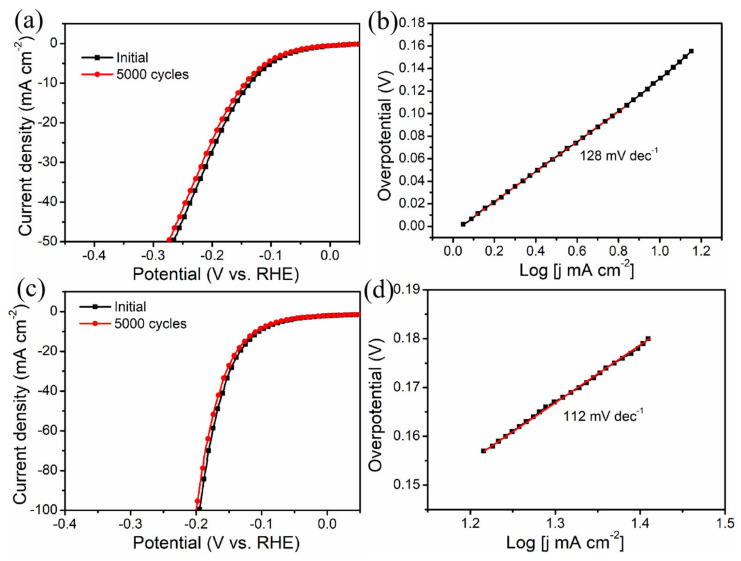
LSV curves of the Ni-CoP_3_-7 NWAs/CC in (**a**) 1.0 M PBS (pH 7) and (**c**) 1.0 M KOH (pH 14), and the corresponding Tafel plots in (**b**,**d**).

The HER electrocatalytic activity of Ni-CoP_3_-7 NWAs/CC in 1.0 M PBS and 1.0 M KOH solutions was also investigated. As shown in Figure 5, the 3D Ni-CoP_3_-7 NWAs/CC needs an overpotential of −139 mV to realize a current density of 10 mA cm^−2^ and shows a low Tafel slope of 128 mV dec^−1^ with excellent durability in 1.0 M PBS solution after 5000 cycles, demonstrating its high-efficiency catalytic activity in 1.0 M PBS solution compared with most of the reported non-noble-metal electrocatalysts (Table S2). Ni-CoP_3_-7 NWAs/CC in a 1.0 M KOH solution (Figure 5) requires an overpotential of -106 mV to achieve a current density of 10 mA cm^−2^ and has a Tafel slope of 112 mV dec^−1^ after 5000 cycles when comparing its electrocatalytic activity to the non-noble-metal electrocatalysts from previous reports (Table S3). The composition, structure and morphology after electrocatalytic test maintain the same to the as-prepared catalysts (Figures S7 and S8). Therefore, the Ni-CoP_3_-7 NWAs/CC exhibits competitive electrocatalytic performance and excellent stability over a large pH range, proving it to be a promising non-noble-metal alternative with great potential for industrial production of hydrogen.

The mechanism for enhanced HER electrocatalytic activity of the Ni-CoP_3_ NWAs is discussed using experimental results and DFT calculations. It can be seen from Figure 3 that the BEs of Co 2*p*_3/2_ (780.0 eV) and P 2*p*_3/2_ (129.7 eV) are slightly shifted from those of metallic Co (778.3 eV) and elemental P (130.1 eV), respectively. This indicates that the Co and P atoms in Ni-CoP_3_-7 NWAs have a partial positive and negative charge, respectively [6,25]. Therefore, a small number of electrons are transferred from Co to P, which is conducive to the catalytic adsorption and desorption of hydrogen atoms. Therefore, the CoP_3_ NWAs have a similar catalytic mechanism as the metal and hydrogenase complex catalysts [36,37].

**Figure 6:**
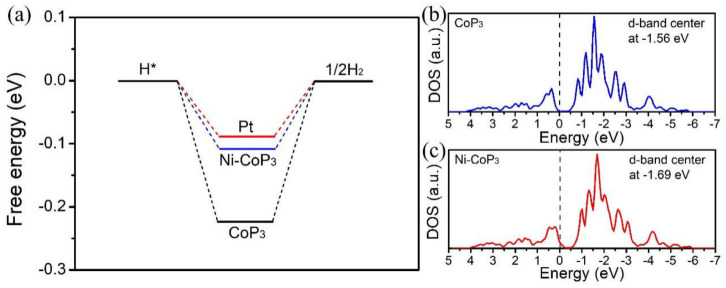
DFT calculations. (**a**) Calculated free-energy diagram of Pt, Ni-CoP_3_, and CoP_3_. (**b**,**c**) Calculated DOS curves for CoP_3_ and Ni-CoP_3_, respectively.

To reveal the intrinsic effect of Ni dopant on the electrocatalytic performance of CoP_3_ and gain new insight into the catalytically active centers, DFT was used to calculate the electronic band structure, adsorption free energy of hydrogen (ΔG_H*_) on Ni dopant and the *d*-band center for hydrogen. The calculation results indicate that it is very beneficial for the Ni substitution of Co in CoP_3_. First, the calculated electronic band structures of CoP_3_ and Ni-CoP_3_ are illustrated in Figure S9. Comparing with CoP_3_, Ni-CoP_3_ has a small bandgap and a larger dispersion of hybridized orbitals in CB and VB since Ni orbitals contribute to the band structures. The larger dispersion of hybridized orbitals and small bandgap lead to a good electrical conductivity. In addition, ΔG_H*_ is a reasonable hydrogen evolution performance descriptor [38,39]. A good HER electrocatalyst requires a value of ΔG_H*_ close to 0 eV to strike a balance between proton transfer and removal of adsorbed hydrogen [40]. The configuration of hydrogen adsorption on CoP_3_ and Ni-CoP_3_ is shown in Figure S10. ΔG_H*_ on the original CoP_3_ (110) surface is −0.23 eV (Figure 6a), consistent with previous studies [41]. Apparently, ΔG_H*_ with Ni doping is −0.13 eV, which is significantly closer to thermal neutrality than that of the original CoP_3_ and comparable to Pt (−0.09 eV), demonstrating a suitable H-binding energy. This generally indicates the optimal HER activity. In KOH solutions, however, in addition to ΔG_H*_, the water dissociation kinetic barrier is another critical factor that reflects the performance of, and sometimes even governs, the entire HER process [42]. A lower kinetic barrier for water dissociation can be effectively realized through synergistic effects [43,44]. Thus, the equilibrium between the hydrolysis barrier and hydrogen BEs determines the optimal HER performance of Ni-CoP_3_ NWAs in 1 M KOH. Furthermore, a previous study [45] suggested that Ni is more conducive to the desorption of OH^−^, while Co exhibits higher performance for the Heyrovsky and Tafel steps in alkaline solutions. Therefore, in the process of hydrogen evolution, the Ni active sites on the surface facilitate hydrolysis dissociation, while Co active sites promote the generation and release of H_2_ [46,47]. To better understand why Ni doping leads to the excellent electrocatalytic hydrogen-evolution activity and how Ni doping alters the electronic energy-band structure of CoP_3_, the DOS was calculated. The *d*-band center is lowered from the Fermi level (Figure 6b) and the *d*-band center decreases from −1.56 eV in CoP_3_ to −1.69 eV after Ni doping, resulting in a decrease of H binding strength [48]. These studies essentially provide the intrinsic finding that the lowering of the *d*-band center reduces the adsorption energy of H, which is conducive to the desorption of H from the electrocatalyst surface for HER.

## 4. Conclusions

In summary, Ni-doped CoP_3_ NWAs integrated on CC are prepared by a simple hydrothermal and high-vacuum phosphorization method. Based on the above results, the Ni-doped CoP_3_ NWAs show excellent HER electrocatalytic activity over a wide range of pH, and the reasons for the improved catalytic HER activity are summarized as follows: (1) the rough surface and NWA structure of CoP_3_ NWAs/CC provide a large specific surface area and high density of active sites; (2) the Ni-doped character of CoP_3_ offers great intrinsic electrocatalytic performance; (3) Ni-doped CoP_3_ NWAs provide good electrical conductivity, which is conducive to rapid electron transfer; and (4) the asymmetric Ni-H and Co–H bonds, as well as a low ΔG_H*_, facilitate the adsorption and desorption of H atoms to produce H_2_. It is therefore clarified in this work that a promising alternative of non-noble-metal electrocatalysts is offered for energy-conversion and energy-storage applications by nearly metallic Ni-doped CoP_3_ NWAs owing to earth-abundant components, facile preparation, extraordinary performance over a wide pH range, and excellent stability.

## Data Availability

The data provided in this study are available on request from The correspondence author.

## Supplementary Materials

The following are available online at https://www.mdpi.com/article/10.3390/nano11061595/s1. Figure S1. EDX spectrum of Ni-CoP3-7 NWAs/CC, Figure S2. (a) Nitrogen adsorption/desorption isotherm and (b) the BJH pore-size distribution curve of CoP3 and Ni-CoP3-7 NWAs, respectively, Figure S3. The dependence of the adsorption time on the amount of absorbed H+ (based on the quality of HCl) on the Ni-CoP3-7 NWAs catalysts in 5 mM aqueous solution, Figure S4. Polarization curves of CC in 0.5 M H2SO4, Figure S5. Cyclic voltammograms in the region of −0.18–0.02 V vs. SCE at various scan rates and the corresponding linear fitting of the capacitive currents vs. scan rates to estimate the double layer capacitance: (a) Ni-CoP3-7 NWAs/CC and (b) CoP3 NWAs/CC; (c) The capacitive currents were measured at −0.08 V vs. SCE plotted as a function of scan rate, Figure S6. TOF curves of CoP3 and Ni-CoP3-7, Figure S7. (a) XRD patterns of Ni-CoP3-7 NWAs/CC and (b), (c), (d) the XRD patterns of Ni-CoP3-7 NWAs/CC electrode after 5000 cycles CV scanning at pH 0, pH 7 and pH 14, respectively, Figure S8. SEM images after 5000 cycles CV scanning at pH 0 (a), pH 7 (b) and pH 14 (c), respectively, Figure S9. Electronic band structure of (a) CoP3 and (b) Ni-CoP3, Figure S10. The configuration of hydrogen adsorption on CoP3 (a) and Ni-CoP3 (b), Table S1. Comparison of HER performance in acid media for CoP3 HSs/CC with other TMPs HER electrocatalysts, Table S2. Comparison of HER performance in neutral media for Ni-CoP3-7 NWAs/CC with other HER electrocatalysts, Table S3. Comparison of HER performance in alkaline media for CoP3 NPAs/CC with other HER electrocatalysts.
