# Supplementary material for: Electronic Structure and d-Band Center Control Engineering over Ni-Doped CoP3 Nanowall Arrays for Boosting Hydrogen Production
Source: Nanomaterials (Basel). 2021 Jun 17;11(6):1595. doi: 10.3390/nano11061595 (PMC8233895; doi:10.3390/nano11061595)
Supplement: Supplementary file 1 [file nanomaterials-11-01595-s001.zip › nanomaterials-1237877-supplementary.pdf]

Supplementary Materials

Article

Electronic Structure and *d*-Band Center Control Engineering over Ni-doped CoP3 Nanowall Arrays for Boosting Hydrogen Production

Jing Qi, Tianli Wu *, Mengyao Xu, Dan Zhou and Zhubing Xiao*

Henan Key Laboratory of Photovoltaic Materials, Henan University, Kaifeng, 475004, China; jingqi_henu@163.com (J.Q.); mengyaoxu_henu@163.com (M.X.); hxzhoud@163.com (D.Z.)

* Correspondence: tianliwu@henu.edu.cn (T.W.); zbxiao@vip.henu.edu.cn (Z.X.)

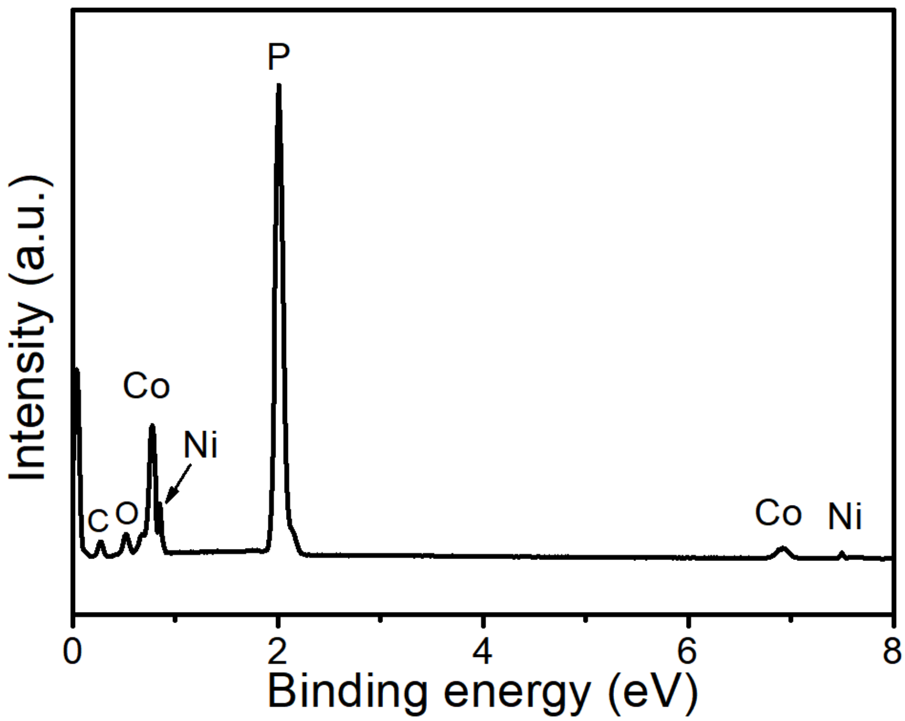

Figure S1. EDX spectrum of Ni-CoP_3-7 NWAs/CC.

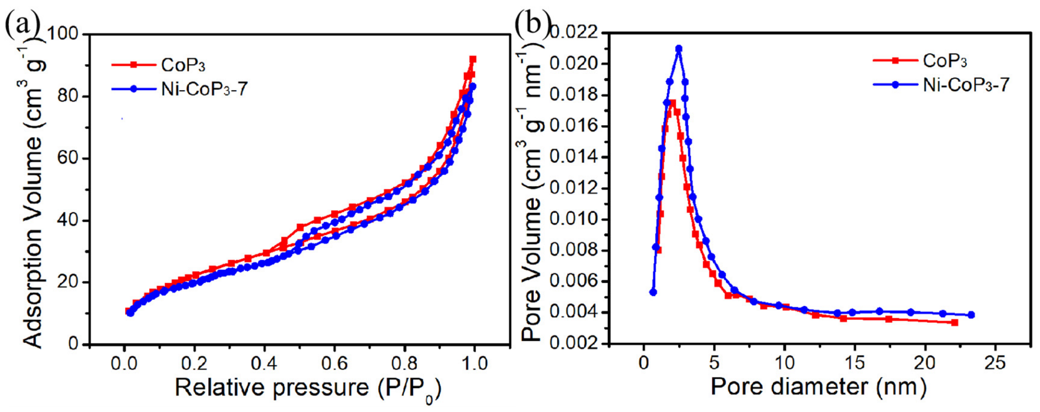

Figure S2. (**a**) Nitrogen adsorption/desorption isotherm and (**b**) the BJH pore-size distribution curve of CoP3 and Ni-CoP_3-7 NWAs, respectively.

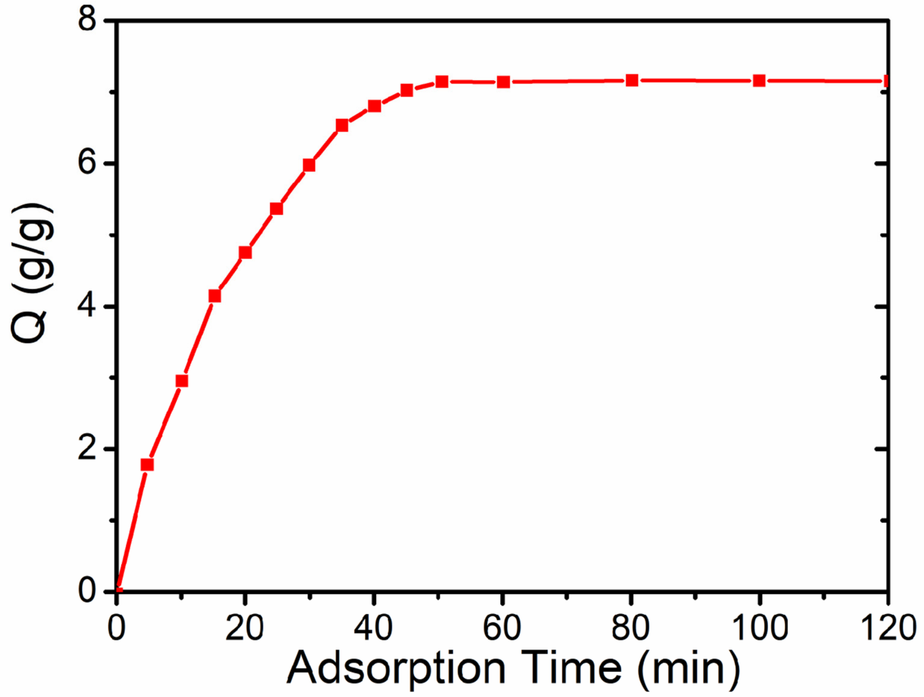

Figure S3. The dependence of the adsorption time on the amount of absorbed H^+ (based on the quality of HCl) on the Ni-CoP_3-7 NWAs catalysts in 5 mM aqueous solution.

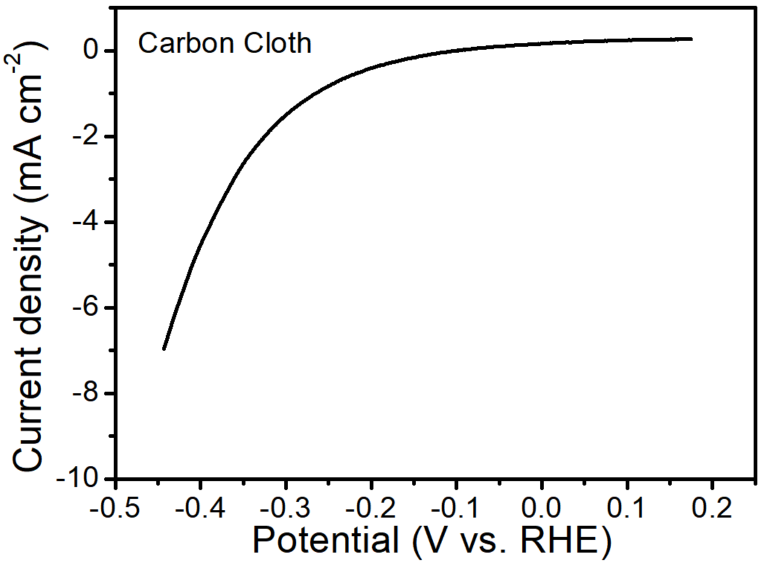

Figure S4. Polarization curves of CC in 0.5 M H_2SO_4.

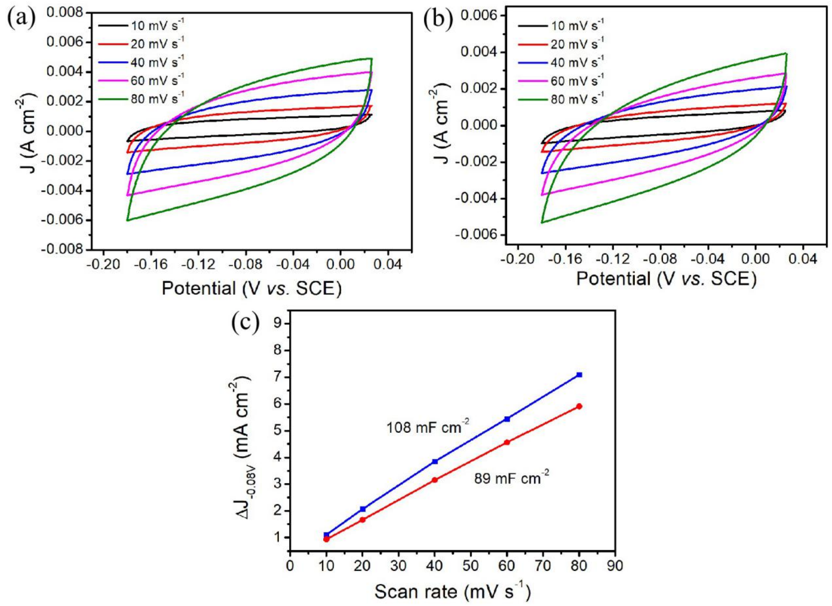

Figure S5. Cyclic voltammograms in the region of -0.18-0.02 V vs. SCE at various scan rates and the corresponding linear fitting of the capacitive currents vs. scan rates to estimate the double layer capacitance: (**a**) Ni-CoP_3-7 NWAs/CC and (**b**) CoP_3 NWAs/CC; (**c**) The capacitive currents were measured at -0.08 V vs. SCE plotted as a function of scan rate.

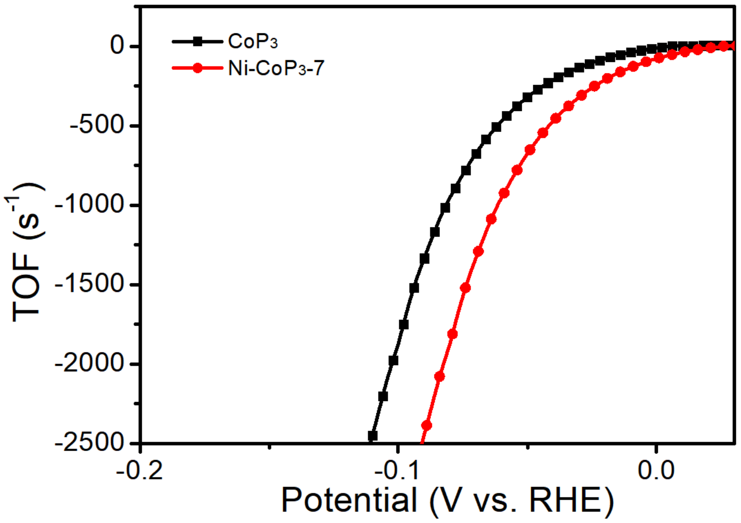

Figure S6. TOF curves of CoP_3 and Ni-CoP_3-7.

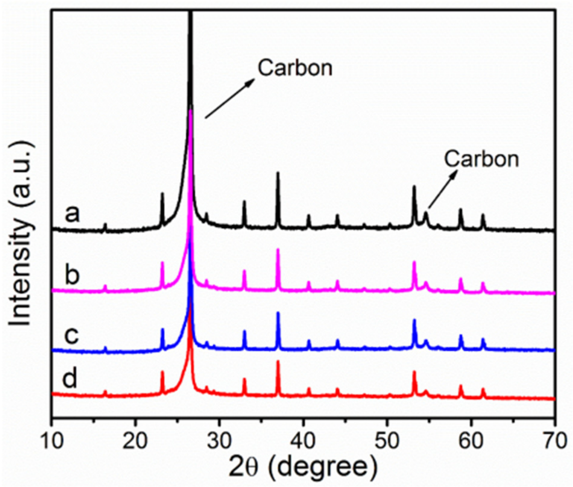

Figure S7. (**a**) XRD patterns of Ni-CoP_3-7 NWAs/CC and (**b–d**) the XRD patterns of Ni-CoP_3-7 NWAs/CC electrode after 5000 cycles CV scanning at pH 0, pH 7 and pH 14, respectively.

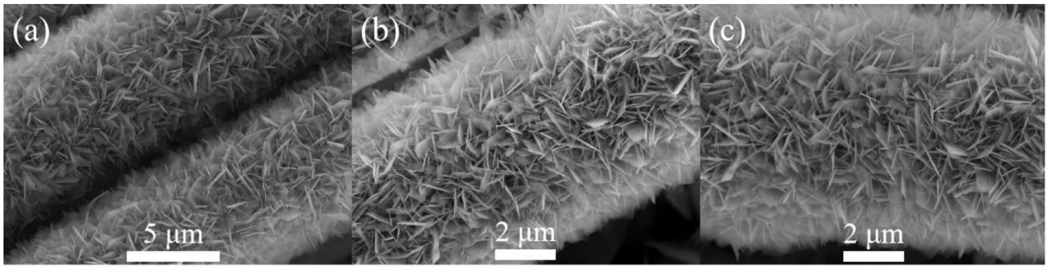

Figure S8. SEM images after 5000 cycles CV scanning at pH 0 (**a**), pH 7 (**b**) and pH 14 (**c**), respectively.

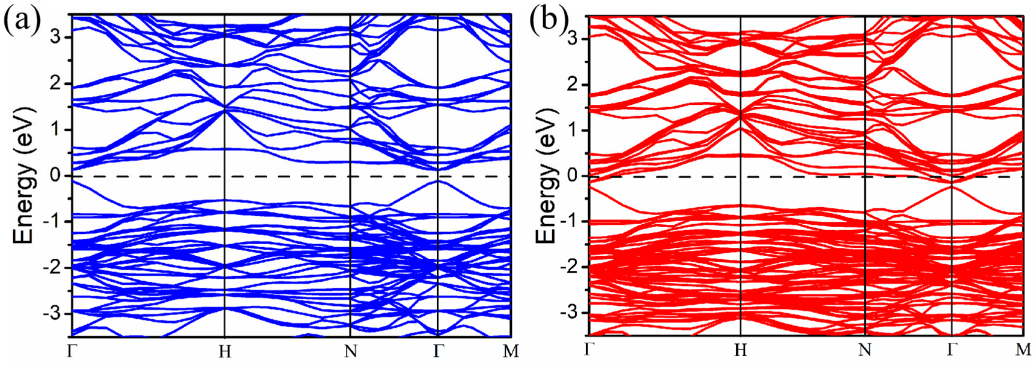

Figure S9. Electronic band structure of (**a**) CoP_3 and (**b**) Ni-CoP_3.

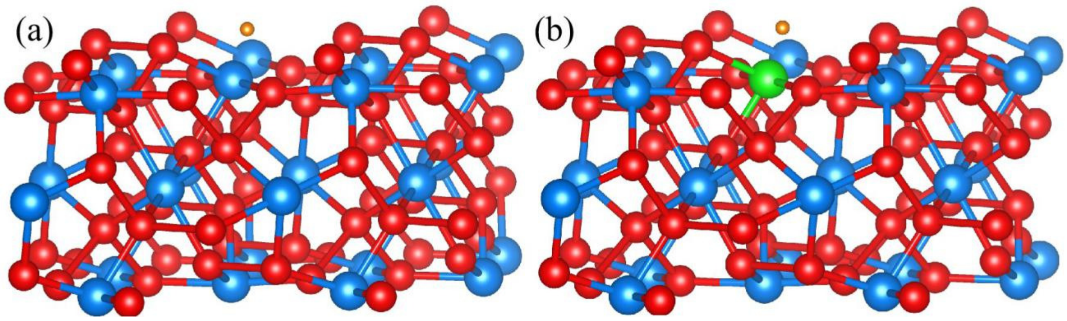

Figure S10. The configuration of hydrogen adsorption on CoP_3 (**a**) and Ni-CoP_3 (**b**).

Table S1. Comparison of HER performance in acid media for CoP_3 HSs/CC with other TMPs HER electrocatalysts.

Catalyst	Onset η (mV)	Current density (*j*, mA cm^{-2})	η at the corresponding *j* (mV)	Exchange current density (mA cm^{-2})	Ref.
**FeP nanosheets**	100	10	240	-	*Chem. Commun.*, 2013, 49, 6656
**FeP NWs/Ti**	38	10	55	0.42	*Angew. Chem. Int. Ed.*, 2014, 53, 12855
**FeP NWs**	-	10	96	0.17	*Chem. Commun.*, 2016, 52, 2819.
**FeP NPs**	38	10	112	-	*Nanoscale*, 2015, 7, 4400
**FeP NAs**	-	10	85	-	*J. Mater. Chem. A*, 2014, 2, 17263
**FeP_2/C**	-	5	500	$1.75*10^{-3}$	*J. Mater. Chem. A*, 2015, 3, 499
**Ni_2P NPs**	-	20	130	$2.7*10^{-3}$	*J. Am. Chem. Soc.*, 2013, 135, 9267
**Ni_2P/graphene**	37	10	102	0.049	*J. Power Sources*, 2015, 297, 45
**Ni_2P/CNs**	40	10	92	-	*J. Power Sources*, 2015, 285, 169
**NiP_2/CC**	-	10	116	0.26	*Nanoscale*, 2014, 6, 13440
**Se doped NiP_2**	-	10	84	-	*ACS Catal.*, 2015, 5, 6355
**Cu_3P NWs**	62	10	143	0.18	*Angew. Chem. Int. Ed.*, 2014, 53, 9577
**MoP Bulk**	50	30	180	0.034	*Energy Environ. Sci.*, 2014, 7, 2624
**MoP NPs**	40	10	125	0.086	*Adv. Mater.*, 2014, 26, 5702
**MoP_2 NPs/Mo**	-	10	143	0.06	*Nanoscale, 2016, 8, 8500*
**MoP_2 nanosheets**	-	10	58	-	*J. Mater. Chem. A*, 2016, 4, 7169
**WP**	50	10	120	-	*Chem. Commun.*, 2014, 50, 11026
**α-WP_2**	54	10	161	0.017	*ACS Catal.*, 2015, 5, 145
**β-WP_2**	56	10	148	0.013	*J. Power Sources*, 2015, 278, 540
**Co_2P**	-	10	95	-	*Chem. Mater.*, 2015, 27, 3769
**CoP/CC**	38	10	67	0.288	*J. Am. Chem. Soc.*, 2014, 136, 7587
**CoP NPs**	-	20	85	-	*Angew. Chem. Int. Ed.*, 2014, 53, 5427
**CoP/CNs**	40	10	122	0.13	*Angew. Chem. Int. Ed.*, 2014, 53, 6710
**Ni-CoP_3 NWAs**	33	10<br>**100**	95<br>**177**	0.217	This work

NPs (Nanoparticles); CNs (Carbon Nanotubes); CC (Carbon Cloth); NWs (Nanowires); NAs (Nanoneedle Arrays); CPs (Concave Polyhedrons)

Table S2. Comparison of HER performance in neutral media for Ni-$CoP_{3\text{-}7}$ NWAs/CC with other HER electrocatalysts.

Catalyst	Current density (j, mA cm^{-2})	Potential at the corresponding j (mV)	Ref.
**Mo_2B**	1	250	*Angew. Chem. Int. Ed.*, 2012, 51, 12703
**Mo_2C**	1	200	*Angew. Chem. Int. Ed.*, 2012, 51, 12703
**Co-S/FTO**	2	83	*J. Am. Chem. Soc.*, 2013, 135, 17699
**$CuMoS_4$**	2	210	*Energy Environ. Sci.*, 2012, 5, 8912
**WP**	10	200	*ACS Appl. Mater. Interfaces*, 2014, 6, 218740
**WP_2**	10	298	*J. Power Sources*, 2015, 278, 540
**MoP_2 NPs/Mo**	10	211	*Nanoscale*, 2016, 8, 8500
**CoP NPs**	2	65	*J. Am. Chem. Soc.*, 2014, 136, 7587
**CoP_3 NPAs**	10	138	*Appl. Surf. Sci.*, 2019, 494, 179-186
**Ni-CoP_3-7 NWAs**	10	129	This work

Table S3. Comparison of HER performance in alkaline media for CoP_3 NPAs/CC with other HER electrocatalysts.

Catalyst	Current density (j, mA cm^{-2})	Potential at the corresponding j (mV)	Ref.
**Ni wire**	10	350	*ACS Catal.*, 2013, 3, 166
**MoB**	10	225	*Angew. Chem. Int. Ed*, 2012, 51, 12703
**NiP_2**	10	102	*Nanoscale*, 2014, 6, 13440
**WP**	10	250	*ACS Appl. Mater. Interfaces*, 2014, 6, 218740
**WP_2**	10	225	*J. Power Sources*, 2014, 136, 7587
**MoP_2 nanosheets**	10	85	*J. Mater. Chem. A*, 2016, 4, 7169
**MoP_2 NPs/Mo**	10	194	*Nanoscale*, 2016, 8, 8500
**CoP nanowires**	10	209	*J. Am. Chem. Soc.*, 2014, 136, 7587
**CoP_3 NPAs**	10	109	*Appl. Surf. Sci.*, 2019, 494, 179-186
**Ni-CoP_3-7 NWAs**	10	102	This work
